# Rosiglitazone increases endothelial cell migration and vascular permeability through Akt phosphorylation

**DOI:** 10.1186/s40360-017-0169-y

**Published:** 2017-08-30

**Authors:** Yun Hyi Ku, Bong-Jun Cho, Min Joo Kim, Soo Lim, Young Joo Park, Hak C. Jang, Sung Hee Choi

**Affiliations:** 1 Department of Internal Medicine, Korea Cancer Center Hospital, Seoul, South Korea; 2 Department of Internal Medicine, Seoul National University Bundang Hospital, Seongnam, South Korea; 3 166 Gumi-dong, Bundang-gu, Seongnam-si, Gyeonggi-do 463-707 Republic of Korea

**Keywords:** Thiazolidinediones, Rosiglitazone, Endothelial cells, Vascular permeability, Edema, Akt

## Abstract

**Background:**

Thiazolidinediones (TZDs), peroxisome proliferator-activated receptor-γ (PPAR-γ) agonists, exhibit anti-inflammatory and antioxidant properties and inhibit endothelial inflammation and dysfunction, which is anti-atherogenic. However, fluid retention, which may lead to congestive heart failure and peripheral edema, is also a concern, which may result from endothelial cell leakage. In the current study, we examined the effects of PPAR-γ agonists on vascular endothelial cell migration and permeability in order to determine its underlying mechanisms.

**Methods:**

We used rosiglitazone and conducted cell migration assay and permeability assay using HUVEC cells and measured vascular permeability and leakage in male C57BL/6 mice.

**Results:**

Rosiglitazone significantly promoted endothelial cell migration and induced permeability via activation of phosphatidylinositol-3-kinase (PI3K) – Akt or protein kinase C (PKC)β. In addition, rosiglitazone increased vascular endothelial growth factor (VEGF) expression and suppressed expression of tight junction proteins (JAM-A and ZO-1), which might promote neovascularization and vascular leakage. These phenomena were reduced by Akt inhibition.

**Conclusions:**

Vascular endothelial cell migration and permeability change through Akt phosphorylation might be a mechanism of induced fluid retention and peripheral tissue edema by TZD.

## Background

Cardiovascular disease is a leading cause of death related to atherosclerosis. Atherosclerosis arises from a series of proinflammatory, proliferative, and procoagulatory processes [1, 2]. Compromised endothelial cell function plays a critical role in the development of atherosclerosis [3]. Endothelial dysfunction can be characterized as an alteration in cell migration and permeability. Endothelial cell migration involves the neovascularization of atherosclerotic plaques and causes plaques to become vulnerable. In addition, newly formed small vessels can provide an entry for inflammatory cells [4]. Under physiological conditions, endothelial cells provide a barrier between blood vessels and tissues [5, 6]. However, inflammatory stimuli can increase endothelial permeability [6, 7] and enable the movement of macromolecules such as vascular endothelial growth factor (VEGF), which can induce neovascularization [8, 9]. It is suggested that endothelial cell migration is induced by angiogenic factors such as VEGF and linked to the phosphatidylinositol-3-kinase (PI3K) – Akt pathway for neovascularization [10–12]. Endothelial cells must adhere to extracellular matrix for their survival [9, 13] and cellular adhesion to matrix is mediated by a few junctional proteins. During the process of atherosclerosis, tight junctions of endothelial cells may loosen, resulting in an increase in vascular permeability [14].

Peroxisome proliferator-activated receptors (PPARs) are nuclear receptor families that have critical functions in lipid homeostasis and peripheral insulin sensitivity [15]. PPAR-γ is essential for adipocyte differentiation [16] and fat storage [17, 18]. It is also the molecular target of the thiazolidinedione (TZD) class of anti-diabetic drugs such as rosiglitazone [19]. PPAR-γ is expressed in endothelial cells [20] and previous preclinical studies have reported that PPAR-γ agonist inhibit endothelial inflammation and dysfunction [21]. In clinical practice, fluid retention such as peripheral edema and congestive heart failure is known as the most common and serious side effect of TZDs and has become the most frequent cause of discontinuation of therapy [22]. Conflicting data have been reported with regard to the mechanism by which TZD acts on vascular endothelial cells.

In endothelial cells, cellular signaling is dependent on two major pathways – PI3K/Akt/mTOR pathway and ERK/MAPK (extracellular signal-regulated kinases/mitogen-activated protein kinases) pathway. TZDs were reported to affect vascular constriction by inhibiting PI3K/Akt pathway [23] and the stimulation of PPAR-γ by TZDs activates the MEPK / ERK pathway and promotes adipogenesis [24]. In addition, the overexpression of PKCβ in endothelial cells has been reported to inhibit Akt phosphorylation and affect PI3K/Akt pathway [25].

In the current study, we hypothesized that one of the thiazolidinediones, rosiglitazone acts positively in terms of smooth muscle cell migration, endothelial cell repair, and vascular inflammation, but negatively affects blood vessel leakage, leading to leakage and increased edema and examined the effect of rosiglitazone on the expression of VEGF and PI3K-Akt signaling in human umbilical vascular endothelial cells (HUVEC) and mice fed a high fat diet (HFD).

## Methods

### Drugs

Rosiglitazone, PI3K inhibitor (LY294002), Akt specific inhibitor, and protein kinase C (PKC) β inhibitor were purchased from Calbiochem (San Diego, CA, USA).

### Cell culture

Primary HUVECs (CC-2519, Cambrex, Walkersville, MD, USA) were cultured in endothelial cell growth medium (EGM)-2 (Cambrex) containing 2% fetal bovine serum (FBS), 0.4% hydrocortisone, 4% hFGF-B, 0.1% VEGF, 0.1% R3-IGF, 0.1% ascorbic acid, 0.1% hEGF, 0.1% GA-1000, and 0.1% heparin. Cells were grown at 37 °C and 5% CO_2_. For the experiment, HUVECs were starved in endothelial cell basal medium (EBM)-2 (Cambrex) supplemented with 0.4% FBS for 24 h. HUVECs of passages 2–9 were used in the experiments.

### Wound healing assay

HUVECs were grown to confluence in 6-well plates (NalgeNunc, Rochester, NY, USA). Each well was scratched with a 1000 μL pipette tip to generate wounds and then starved as described above with or without rosiglitazone. At least 3 images were taken using a microscope and the distance moved was measured to estimate cell migration.

### Transwell migration assay

The modified Boyden chamber assay was performed using a Transwell system (Corning, Rochester, NY, USA). The insert had a polycarbonate membrane with 8 μm pores which enable migration of cells. Each insert was coated with a 10 μg gelatin solution and 10^4^ HUVEC cells in starvation medium were loaded onto the insert. Each well was filled with 500 μL of starvation medium with or without rosiglitazone and inserts were placed in 24-well plates. After 24 h, the upper surface of the inserts was swabbed with a cotton-tipped applicator for removal of non-migrating cells. Inserts were fixed in methanol for 10 min and stained with 1% crystal violet for 2 h. The number of cells that had moved was counted to estimate cell migration.

### Transwell monolayer permeability assay

HUVECs (8 × 105/well) were seeded on 2 μm PET Transwell inserts (Falcon, USA) and grown to confluence with EGM-2. The confluence, integrity, and uniformity of the endothelial cell monolayer were examined microscopically. Before performing the experiment, cells were starved for 24 h with rosiglitazone and inhibitors. To determine the permeability through the cell monolayer, Evans Blue (EB) bound to 0.1% bovine serum albumin (BSA) in phosphate-buffered saline (PBS) was loaded onto the upper chamber. EB could leak through intercellular spaces without junction or endothelial cell fenestration into the lower chamber. After 1 h, the amount of EB in the lower chamber was determined by spectrophotometer (620 nm). Experiments were performed in triplicate and repeated multiple times.

### Immunofluorescence staining

Cells were plated into 8-well slide chambers (Sonic Seal Slides, NalgeNunc). Cells were grown with EBM-2 and reagents for 24 h, respectively. Then, cells were fixed in absolute methanol and washed with PBS containing 0.4% Tween-20. After washing, cells were blocked with PBS containing 10% goat serum, followed by incubation with rabbit polyclonal anti-phospo-Akt (ser473), anti-VEGF, anti-junction adhesion molecule-A (JAM-A), anti-ZO-1, and anti-VEGF antibodies overnight. Cells were processed using FITC-conjugated bovine anti-rabbit antibody. DAPI (1 μg/mL) was used for nuclei counter staining. Images were acquired on a Zeiss or Leitzorthoplan microscope (Leica Inc., Wetzlar, Germany) or a Leica DMIRBE inverted epifluorescence Nomarski microscope with Leica TCS NT confocal laser optics.

### Western blot

Cells were harvested and solubilized in cell lysis buffer (Cell Signaling, Beverly, MA, USA) for 30 min at 4 °C. Protein concentration was measured using a Bradford protein assay kit (BioRad, Hercules, CA, USA). The same amounts of proteins from whole cell lysates were subjected to SDS polyacrylamide gel electrophoresis and transferred onto methanol-treated PVDF membranes (Millipore Co, Bedford, MA, USA). The membrane was immunoblotted with antibodies against phospho-Akt, VEGF, JAM-A (Santa Cruz, CA, USA), phospho-PKCβ, ZO-1 (Abcam, Cambridge, MA, USA), and β-actin (Sigma-Aldrich, St. Louis, MO, USA), respectively, overnight at 4 °C. The membranes were washed and incubated for 1 h at room temperature with HRP-conjugated secondary antibodies. After extensive washing, the bands were detected using an enhanced chemiluminescence (ECL) kit (Santa Cruz).

### siRNA transfection

Endogenous Akt activity was inhibited using an RNA interference technique. HUVECs were grown to 80% confluence in a 6-well plate and 40 nM of nonspecific or Akt-1 specific small interfering RNA (siRNA) (Santa Cruz) were transfected into HUVECs according to the manufacturer’s instructions with Lipofectamine 2000 reagent (Invitrogen).

### Animals

**Fig. 1:**
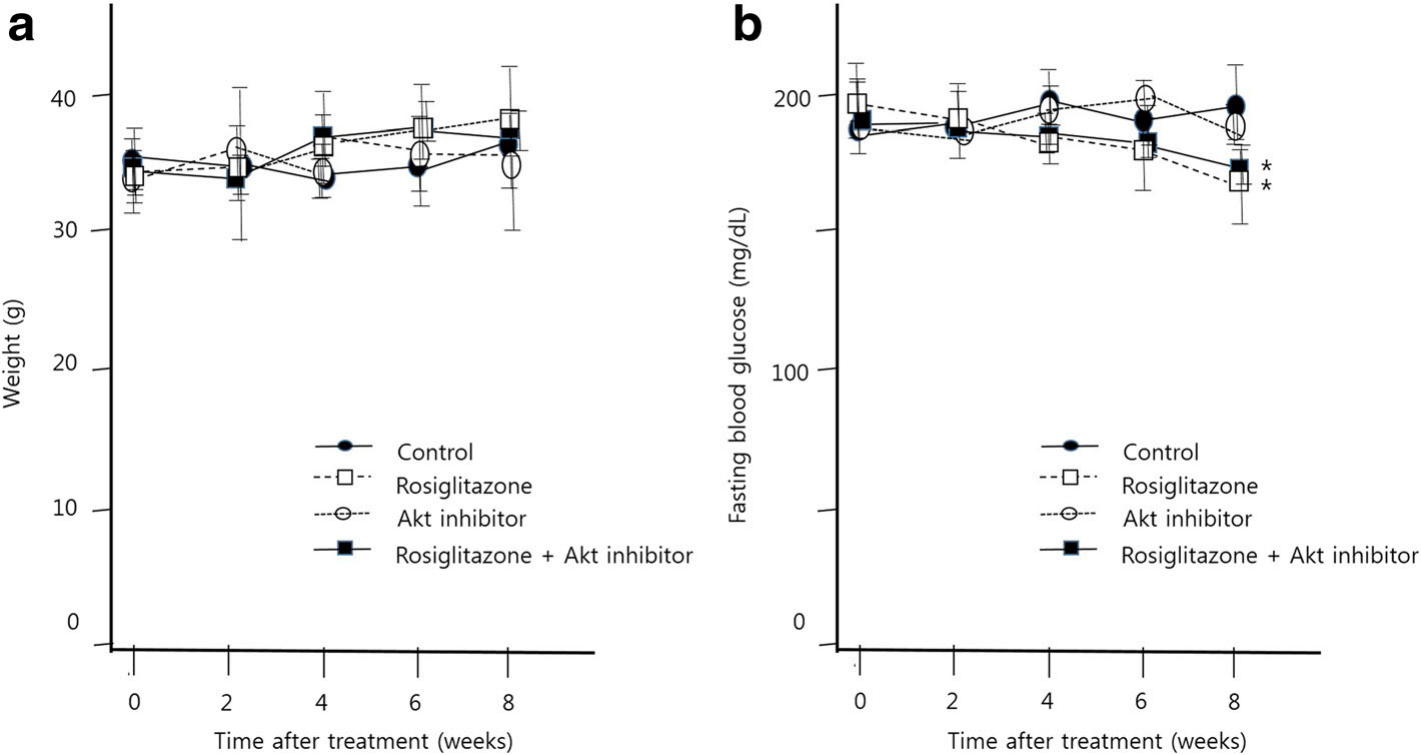
Effects of rosiglitazone or AKT inhibitor on body weight and blood glucose level. **a** Body weight in control, rosiglitazone, Akt inhibitor and rosiglitazone + Akt inhibitor groups. There was a tendency for body weight to increase with rosiglitazone treatment, but there was no statistically significant difference between the four groups (P = NS). **b** Blood glucose levels in four groups. There was a statistically significant decrease in blood glucose in the rosiglitazone group (*P < 0.05)

Male C57BL/6 mice obtained from the central laboratory animal facility (Seoul, Korea) were grown to 6 weeks of age and fed a high fat diet (HFD). At 8 weeks of age, Ed – the C57BL/6 mice were divided into 4 groups; control group (intraperitoneal injection with normal saline; *n* = 6), rosiglitazone group (5 mg/kg/day rosiglitazone [26–29] in diet and injection with normal saline; *n* = 6), Akt inhibitor group (intraperitoneal injection 2 times a week with 0.15 mg/kg [30, 31] of Akt inhibitor with normal saline; *n* = 6), rosiglitazone with Akt inhibitor group (5 mg/kg/day rosiglitazone in diet and injection 2 times a week with 0.15 mg/kg of Akt inhibitor with normal saline; *n* = 6) for 8 weeks. The body weight was approximately 35 g in the control group and tended to increase by rosiglitazone, but this was not statistically significant (Fig. 1a). Fasting glucose level was 190–200 mg/dL in control group, whereas 175–180 mg/dL in rosiglitazone group (Fig. 1b). Akt inhibitor did not make a difference in body weight and blood glucose, compared to control (Fig. 1b). Mice were anesthetized intraperitoneally with a combination of ketamine (70 mg/kg) and xylazine (7 mg/kg; Yuhan Corp, Bayer Korea). All animals were handled in compliance with the Guide for Experimental Animal Research of the Laboratory of Clinical Research Institute, Seoul National University Bundang Hospital. No irritability or restlessness was observed after administration of drug or vehicle. No noticeable adverse effects (e.g., respiratory distress, abnormal locomotion, or catalepsy) were observed in any animals. All mice were maintained in plastic cages in an air-conditioned room at 22 ± 2 °C and 55 ± 10% humidity.

### Measurement of vascular permeability with Evans blue dye

EB dye does not bind covalently to plasma albumin, a marker for proteins around 67 Ed –kDa. Under deep anesthesia, EB dye was injected into a limb artery of male C57BL/6 mice. After 30 min, perfusion with normal saline was administered for 2 min in each mouse to clear the dye from the left ventricle. The main arteries were removed immediately and then frozen in OCT compound. Remaining tissues were flash frozen with liquid N_2_ and kept at −80 °C. The eyes were marked for orientation, enucleated, and placed in 4% paraformaldehyde for 3 to 24 h. Lenses were removed and peripheral retinas were cut up to allow flat mounting with glycerol-gelatin. Images were acquired on a Zeiss or Leitzorthoplan microscope (Leica Inc., Wetzlar, Germany) or a Leica DMIRBE inverted epifluorescence Nomarski microscope with Leica TCS NT confocal laser optics.

### Statistical analysis

Results are reported as the mean ± SEM. Mean values were compared between the drug treatment and control groups by analysis of variance (ANOVA) with a post hoc test, and *P* < 0.01 was considered statistically significant.

## Results

### Rosiglitazone promoted the migration of HUVECs

**Fig. 2:**
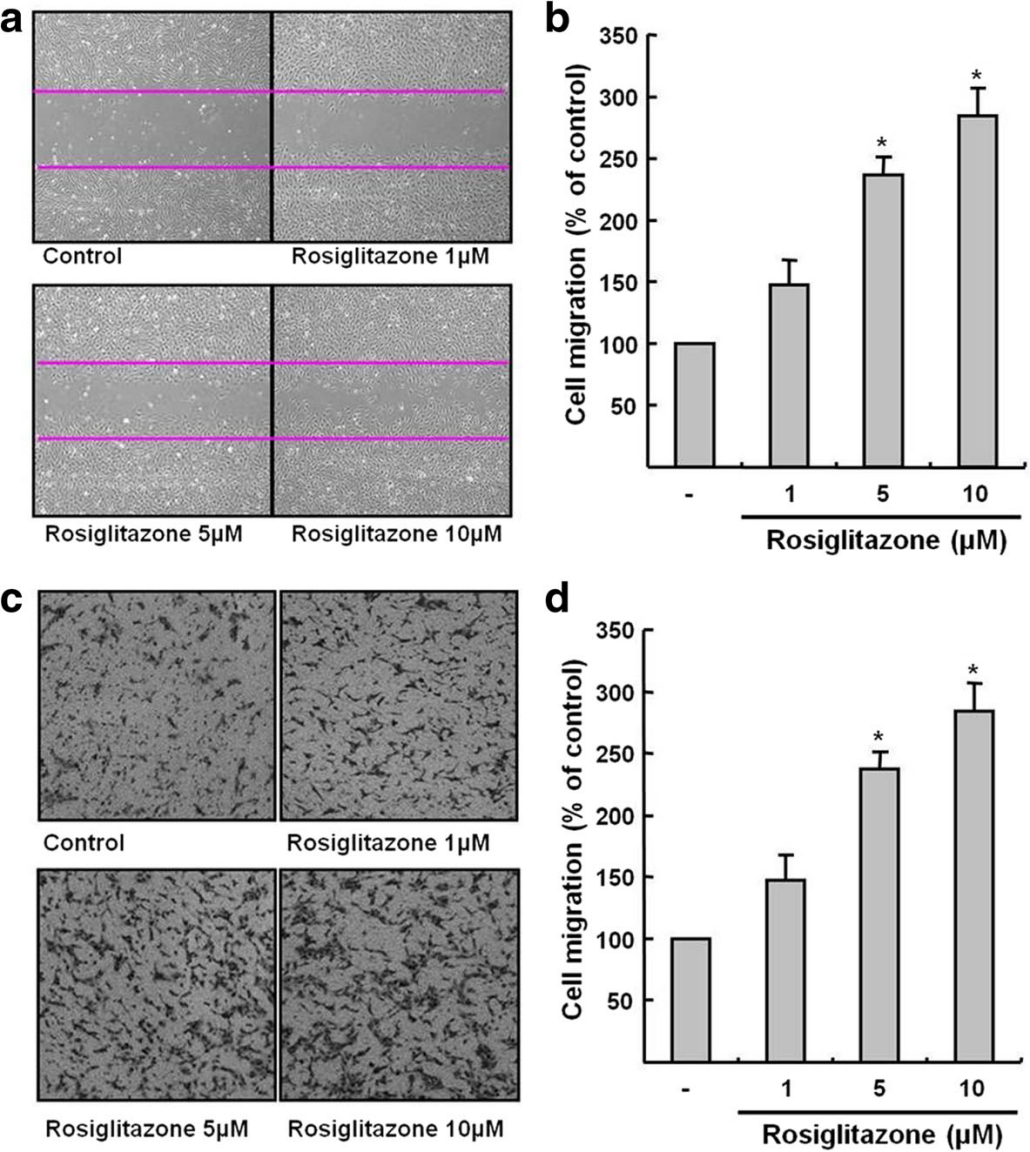
Rosiglitazone promoted the migration of HUVECs. **a** and **b** Cell migration was assessed by a wound healing assay. Representative micrographs at × 400 magnification (**a**) and the distance moved of cells (**b**) without or with rosiglitazone (1, 5, or 10 μM) for 24 h are shown. **c** and **d** Cell migration was assessed by a Transwell migration assay. Representative micrographs at × 400 magnification (**c**) and the number of cells that moved without or with rosiglitazone (1, 5, or 10 μM) for 24 h are shown. Data are represented as % of control. (*n* = 4; * *p* < 0.01 compared with control)

HUVECs were treated with rosiglitazone (1, 5 or 10 μM) for 24 h. In a wound healing assay, 5 and 10 μM of rosiglitazone increased cell migration compared with control (237 ± 17% and 284 ± 22%, respectively; Fig. 2a and b). In a Transwell migration assay, 5 and 10 μM of rosiglitazone also increased cell migration (169 ± 11% and 195 ± 15%, respectively; Fig. 2c and d) in a dose-dependent manner.

### PI3K, Akt, or PKCβ inhibitor suppressed the rosiglitazone-induced endothelial cell migration and permeability

**Fig. 3:**
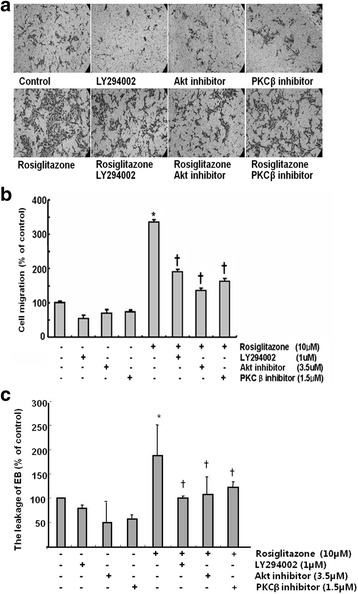
PI3K, Akt, or PKCβ inhibitor suppressed the rosiglitazone-induced endothelial cell migration and permeability. **a** HUVECs were treated with rosiglitazone (10 μM) with or without PI3K inhibitors LY294002 (1 μM), Akt inhibitor (3.5 μM), or PKCβ inhibitor (1.5 μM) simultaneously. Representative micrographs at × 400 magnification. **b** The numbers of cells that moved were counted. Data are represented as % of control. **c** HUVECs were treated with rosiglitazone (10 μM) with or without PI3K inhibitors LY294002 (1 μM), Akt inhibitor (3.5 μM), or PKCβ inhibitor (1.5 μM) simultaneously. The leakage of EB bounded to albumin from the upper to lower chamber was measured. Data are represented as % of control. (*n* = 4; * *p* < 0.01 compared with control, † *p* < 0.01 compared with wells treated with rosiglitazone only)

We examined the effect of PI3K, Akt, or PKCβ inhibitor on rosiglitazone-induced cell migration. HUVECs were treated with rosiglitazone (10 μM) and inhibitors for 24 h. None of the inhibitors affected cell migration without rosiglitazone. However, rosiglitazone induced significantly increased cell migration and all inhibitors suppressed rosiglitazone-induced cell migration (Fig. 3a). LY294002 (PI3K inhibitor), Akt inhibitor, and PKCβ inhibitor decreased rosiglitazone-induced cell migration by 56, 59, and 57%, respectively (Fig. 3b).

We also tested the effect of rosiglitazone on endothelial cell permeability. HUVECs were treated with rosiglitazone (10 μM) and inhibitors for 24 h. In a Transwell monolayer permeability assay, none of the inhibitors affected cell permeability without rosiglitazone, however, rosiglitazone increased cell permeability by 191 ± 54% compared with control and all inhibitors which blocked rosiglitazone-induced cell permeability. The leakage of EB was decreased by 50, 53, and 61%, respectively (Fig. 3c).

These results suggest that rosiglitazone induced an increase in migration and permeability of HUVECs through activation of PI3K/Akt or PKCβ.

### Rosiglitazone activated PI3K, Akt, and PKCβ

**Fig. 4:**
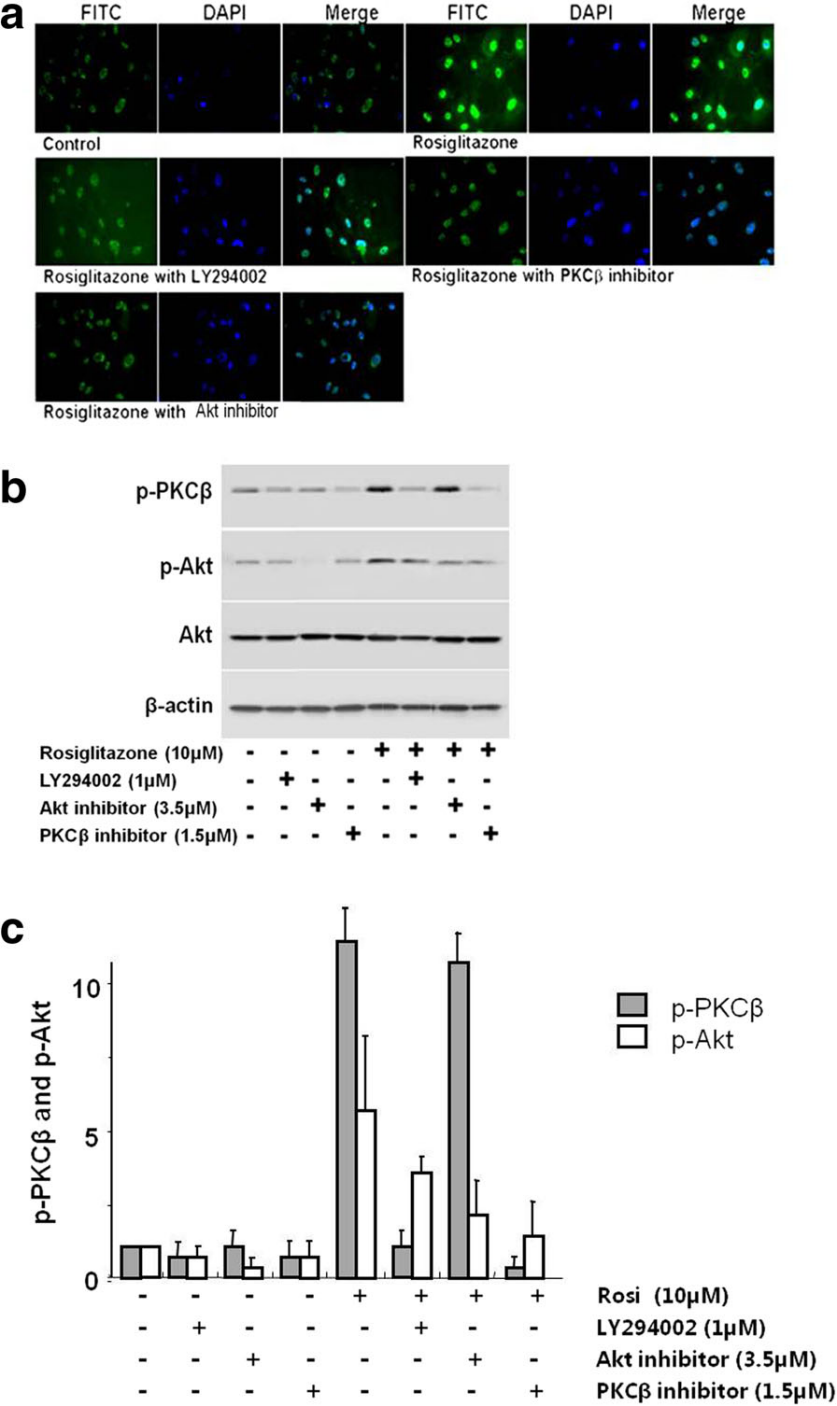
Rosiglitazone activated the phosphorylation of Akt and PKCβ. **a** HUVECs were treated with rosiglitazone (10 μM) with or without PI3K inhibitors LY294002 (1 μM), Akt inhibitor (3.5 μM), or PKCβ inhibitor (1.5 μM) simultaneously. Cells were stained with anti-phospho-Akt antibody. **b** and **c** Immunoblot analysis for the phosphorylation of Akt and PKCβ (*n* = 4)

We found that rosiglitazone-induced endothelial cell migration and permeability was related to PI3K, Akt, and PKCβ activity. To confirm the role of rosiglitazone on Akt activity, we examined the phosphorylation of Akt in rosiglitazone-treated HUVECs. Immunofluorescence staining was performed with anti-phospho-Akt antibody. Rosiglitazone increased the phosphorylation of Akt, which is substantially abundant in the nucleus. However, treatment with LY294002, Akt inhibitor, or PKCβ inhibitor with rosiglitazone resulted in reduced phosphorylation of Akt (Fig. 4a). Western blot analysis also showed that rosiglitazone dramatically increased the phosphorylation of PKCβ and Akt. LY294002 and PKCβ inhibitor remarkably reduced the phosphorylation of Akt and PKCβ, but Akt inhibitor did not affect the phosphorylation of PKCβ (Fig. 4b and c).

### Rosiglitazone increased VEGF expression

**Fig. 5:**
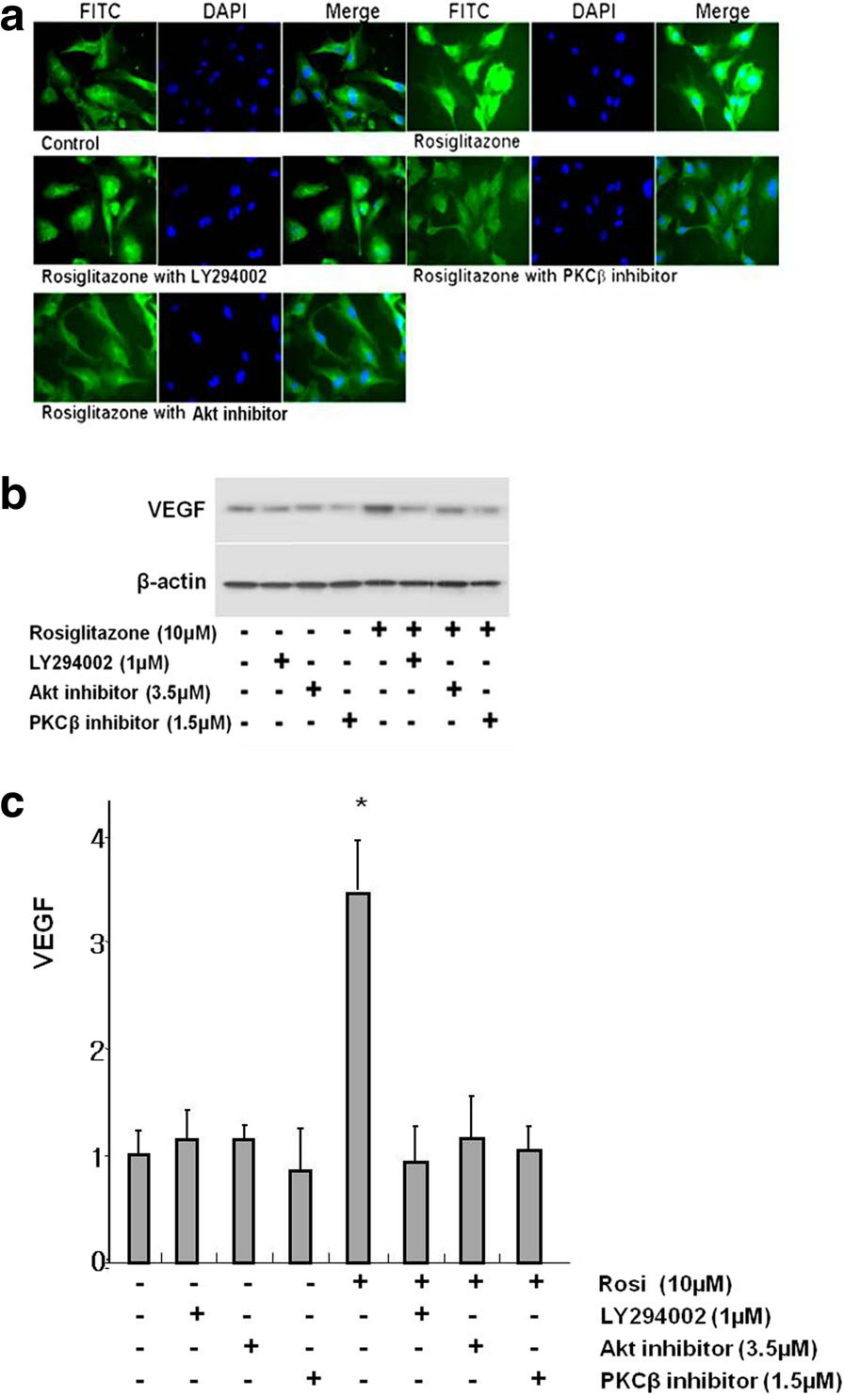
Rosiglitazone increased VEGF expression. **a** HUVECs were treated with rosiglitazone (10 μM) with or without PI3K inhibitors LY294002 (1 μM), Akt inhibitor (3.5 μM), or PKCβ inhibitor (1.5 μM) simultaneously. Cells were stained with anti-VEGF antibody. **b** and **c** Immunoblot analysis for the expression of VEGF (*n* = 4)

VEGF is a potent growth factor for angiogenesis. As shown in Fig. 5a, rosiglitazone significantly increased VEGF expression compared with controls and it was suppressed by LY294002, Akt Inhibitor, and PKCβ inhibitor. Western blot analysis also showed that rosiglitazone markedly increased VEGF and LY294002, Akt Inhibitor or PKCβ inhibitor decreased rosiglitazone-induced VEGF expression (Fig. 5b and c).

### Rosiglitazone suppressed the expression of tight junction proteins

**Fig. 6:**
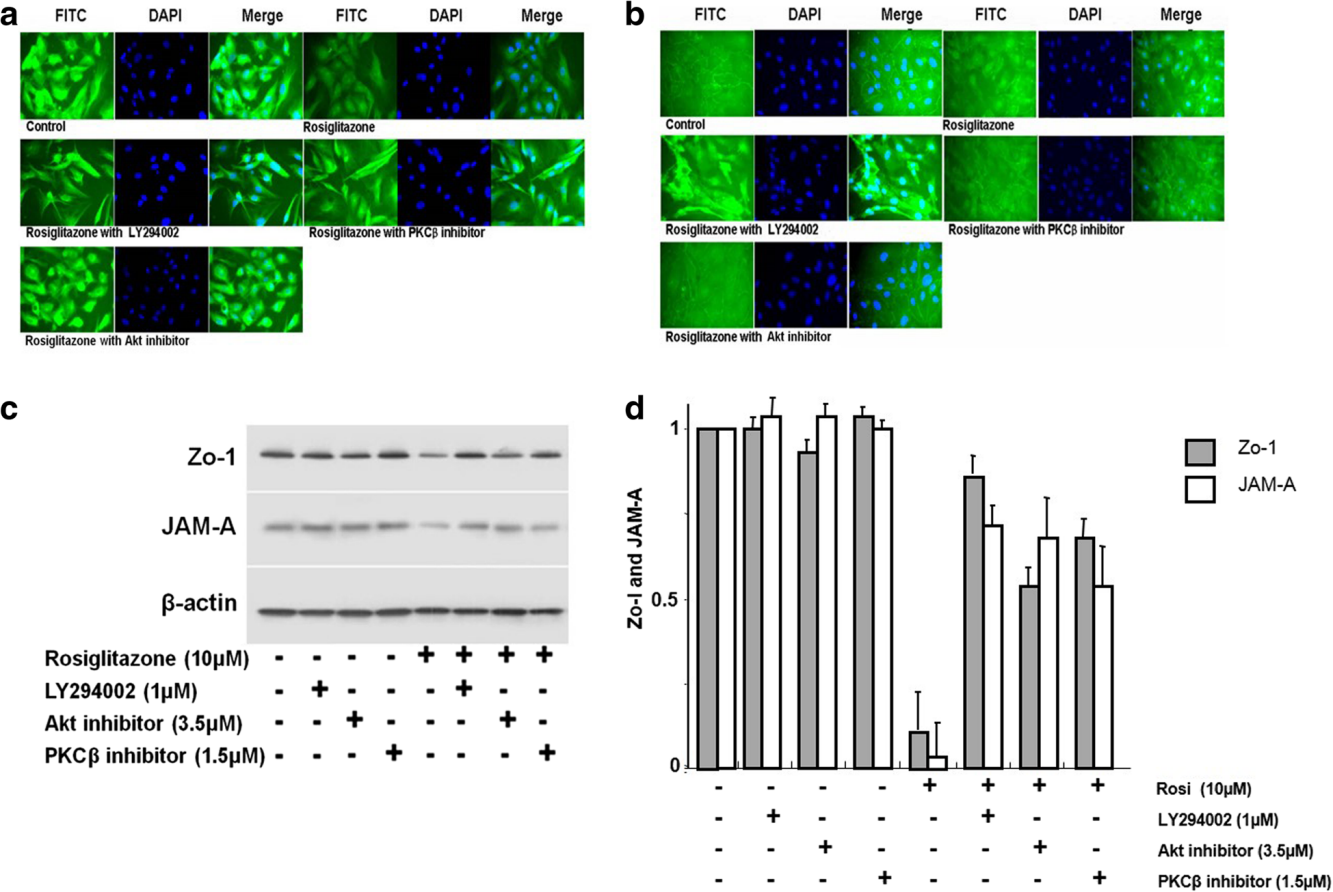
Rosiglitazone suppressed the expression of tight junction proteins. **a** HUVECs were treated with rosiglitazone (10 μM) with or without PI3K inhibitors LY294002 (1 μM), Akt inhibitor (3.5 μM), or PKCβ inhibitor (1.5 μM) simultaneously. Cells were stained with JAM-A antibody. **b** Cells were stained with ZO-1 antibody. **c** and **d** Immunoblot analysis for the expression of JAM-A and ZO-1 (*n* = 4)

Intercellular tight junctions are an important structure in endothelial cells. To understand the effect of rosiglitazone on intercellular junctions, we examined the expression and distribution of two junction proteins, JAM-A and ZO-1 in HUVEC monolayer using immunofluorescence staining (Fig. 6a and b). In un-stimulated cells, JAM-A and ZO-1 were highly expressed on the cell surface and concentrated at cell-cell junctions. Treatment with rosiglitazone resulted in decreased expression of JAM-A and ZO-1 and LY294002, Akt inhibitor, or PKCβ inhibitor recovered the expression. The results of western blot analysis were in accordance with these results (Fig. 6c and d).

### siRNA against Akt-1 inhibits rosiglitazone-induced cell migration

**Fig. 7:**
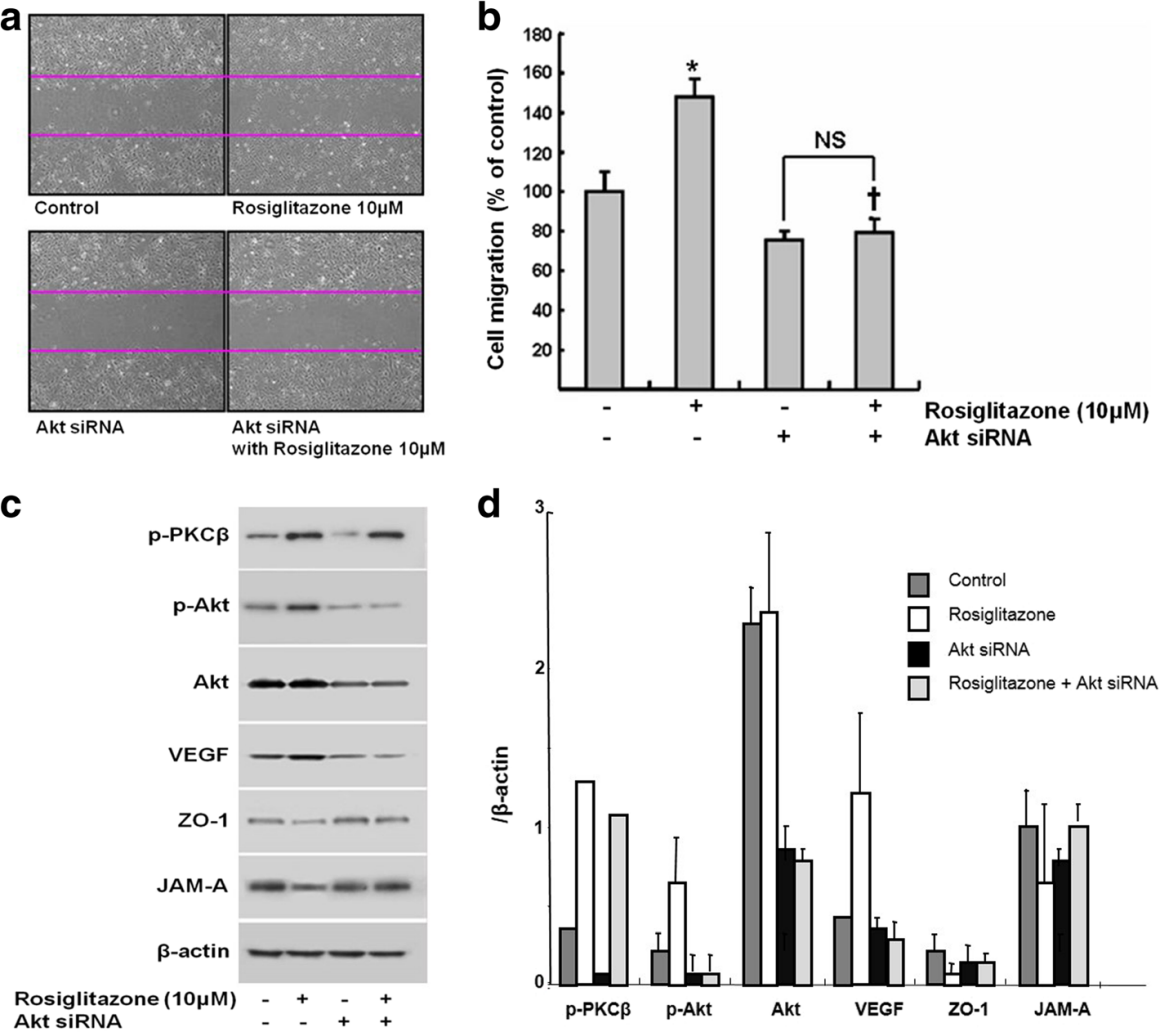
siRNA against Akt-1 inhibits rosiglitazone-induced cell migration. **a** HUVECs transfected with Akt-1 specific siRNA or nonspecific siRNA (control) were treated with rosiglitazone (10 μM) and cell migration was assessed by a wound healing assay. Representative micrographs at × 400 magnifications (**a**) and the distance moved of cells (**b**) (*n* = 4, * *p* < 0.01 compared with control, † *p* < 0.01 compared with control treated with rosiglitazone, p = NS, between Akt siRNA treated groups). **c** and **d** Immunoblot analysis for the expression of phospho-Akt, phosphor-PKC β, VEGF, JAM-A, and ZO-1 (*n* = 4)

To examine the effect of Akt signaling on rosiglitazone-induced cell migration, endogenous Akt was suppressed using siRNA. HUVECs transfected with Akt-1 specific siRNA or with non-targeting siRNA were compared. Transfected cells were treated with rosiglitazone and cell migration was assessed using a wound healing assay (Fig. 7a). Akt siRNA inhibited rosiglitazone induced cell migration by 47 ± 7% compared with control (Fig. 7b). In western blot analysis, the transfection of Akt-1 siRNA effectively reduced endogenous expression of Akt (Fig. 7c and d) and decreased VEGF expression, which was increased by treatment with rosiglitazone and the expression of JAM-A and ZO-1 was also reversed. However, there was no remarkable change in the phosphorylation of PKCβ.

### Rosiglitazone increased neovascularization and vascular leakage in retina and aorta

**Fig. 8:**
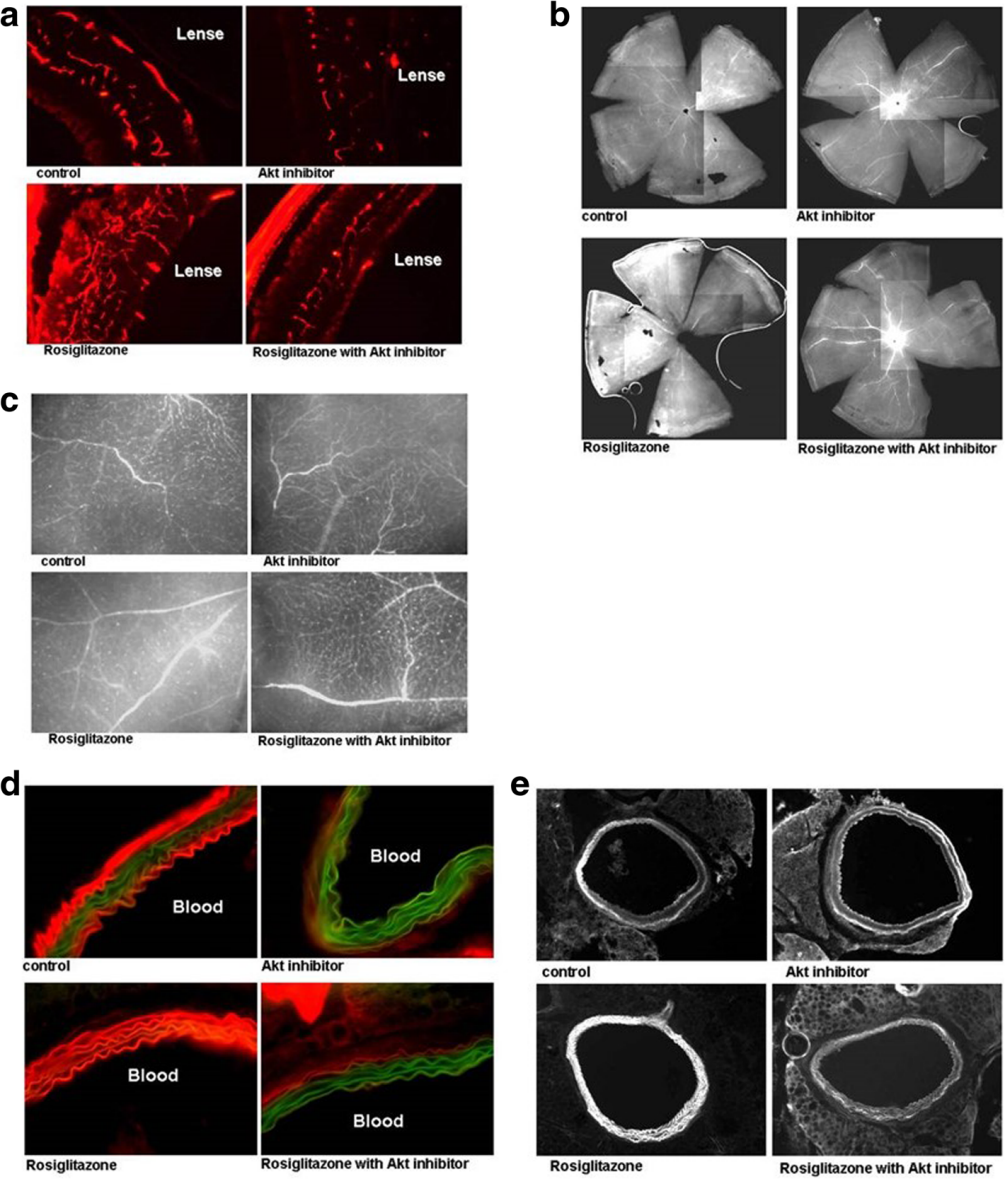
Rosiglitazone increased neovascularization and vascular leakage in retina and aorta. Mice were given HFD with or without rosiglitazone (5 mg/kg/day) and injected intraperitoneally with or without Akt inhibitor (0.15 mg/kg, 2 times in a week) for 8 weeks (*n* = 6/group). **a** Typical photographs from Evans blue-albumin staining of retina whole-mount from mice. **b** Microscopic images of retinal flat-mounts and enlarged area. **c** Microscopic images after injection of Akt inhibitor. **d** Microscopic image of aortic flat-mounts. **e** Microscopic images of the enlarged aortic area

We found that rosiglitazone increased VEGF expression in vitro. We then performed EB staining to examine the effect of rosiglitazone on neovascularization in retina of mice. Administration of rosiglitazone in HFD-fed mice resulted in significantly increased neovascularization (Fig. 8a) and vascular leakage (Fig. 8b) in retina. To clarify the role of Akt in rosiglitazone-mediated retinal neovascularization, Akt inhibitor was injected intraperitoneally. Development of retinal neovascularization by rosiglitazone was effectively suppressed by Akt inhibitor (Fig. 8c). In aorta of mice, rosiglitazone also increased vascular leakage, however injection of Akt inhibitor with rosiglitazone inhibited vascular leakage (Fig. 8d and e).

## Discussion

Atherosclerosis is the primary cause of mortality in patients with type 2 diabetes [32]. Vascular smooth muscle cell (VSMC) proliferation contributes significantly to intimal thickening in atherosclerosis, leading to development of cardiovascular diseases [32]. PPAR- γ agonists such as rosiglitazone have been suggested to inhibit growth and migration of VSMC, with potential favorable effects on atherosclerosis [33]. Peripheral edema formation is a well-known side effect of rosiglitazone. The precise mechanism of this adverse effect is unclear [22], but may involve a number of factors, notably VEGF, nitric oxide, and protein kinase C, etc. [34]. We focused on vascular endothelial cells, not VSMC and supposed that the change of vascular endothelial cell migration and permeability was an important factor which attributed to peripheral edema formation of TZD.

Vascular endothelial cells are activated by physiological agonists and stress stimuli. When activated, endothelial cells release inflammatory mediators like interleukin-1, tumor necrosis factor-α, etc. and induce adhesion molecules like JAM-A and ZO-1, resulting in endothelial dysfunction and increased vascular permeability, particularly atherosclerosis and cardiovascular diseases [35]. PPAR- γ agonists can reduce the activation and inflammation of endothelial cells [36–39]. PPAR-γ agonist inhibits endothelial inflammation by suppressing inflammatory gene expression and therefore improves endothelial dysfunction [21].

In our study, rosiglitazone act positively in terms of smooth muscle cell migration, endothelial cell repair, and vascular inflammation, but negatively affects blood vessel leakage, leading to leakage and increased edema. It appears to be dependent on the PI3K/Akt pathway. Endothelial cell migration is one of the main contributors to the progression of atherosclerotic lesion formation. In our data, rosiglitazone increased endothelial cell migration compared with control in HUVECs. The effects of PPAR-γ agonists on endothelial cell migration are debatable. Goetze et al. [40] reported that PPAR-γ activators including troglitazone and ciglitazone inhibit VEGF-induced endothelial cell migration in HUVECs. Others reported that PPAR-γ activation inhibits VEGF-induced angiogenesis and EC proliferation and migration [41, 42]. By contrast, some authors suggested that PPAR-γ agonists increased cell migration, like our results. Ciglitazone elevated the expression of VEGF mRNA and protein and increased cell viability and migration in breast cancer cells [43]. In human, another PPAR-γ, pioglitazone increased the number and endothelial progenitor cell migratory function in patients with coronary artery disease [44].

VEGF promotes angiogenesis, increases macrophage levels in peripheral blood, and enhances plaque progression, which leads to atherosclerosis and cardiovascular disease [45].

In our experiments, rosiglitazone increased VEGF expression in HUVECs. This observation is in agreement with recent findings. Myofibroblasts have been shown to up-regulate VEGF via oxidized low-density lipoprotein-mediated PPAR-γ activation [46], and bovine articular chondrocytes exhibit a PPAR-γ dependent biosynthesis of VEGF [47]. Other PPAR-γ agonists, troglitazone and ciglitazone, induced VEGF mRNA and protein expression from cultured keratinocytes [48]. However, the effects of rosiglitazone or other PPAR-γ agonists on VEGF expression differed under various conditions.

Rosiglitazone inhibited VEGF expression under the high glucose condition [49], and rosiglitazone failed to induce VEGF expression in keratinocytes [48]. In human Ed – leukocytes, rosiglitazone inhibited VEGF secretion by 63.7% [50]. The cause of these differences in VEGF expression in response to rosiglitazone according to cell types is still unresolved.

In our experiments, rosiglitazone increased endothelial cell permeability and suppressed tight junction proteins as well as migration, which occurred through the Akt dependent pathway. Increased endothelial permeability was observed in atherosclerosis. Low density lipoproteins (LDL) can enter the vascular wall and become oxidized-LDL more easily. At the same time vascular inflammation exacerbates endothelial dysfunction [21]. Oxidized LDLs upregulate VEGF expression in macrophages and endothelial cells through activation of PPAR-γ [32]. VEGF has been recognized as an angiogenic factor that induces vascular permeability. Gavard J et al. reported that VEGF stimulation promotes the beta-arrestin2-dependent endocytosis of VE-cadherin, a key endothelial cell adhesion molecule, disrupting the endothelial barrier function [51]. In HUVECs, both decrease of tight junction protein and increase of VEGF by rosiglitazone together contribute to increased vascular permeability.

There are few data regarding the effects of rosiglitazone on tight junctional proteins. In human nasal epithelial cells, rosiglitazone enhanced the barrier function and upregulation of tight junction molecules including claudin-1, claudin-4, occludin, and tricellulin. In addition, PPAR-γ agonist induced the activity of phospho-PKC. PPAR-γ agonist upregulates the barrier function of tight junctions of human nasal epithelial cells via a PKC signaling pathway [52]. PPAR-γ agonists have protective effects against HIV-induced disruption of brain endothelial cells. HIV reduced junctional protein such as JAM-A and ZO-1, overexpression of PPAR-α or PPAR-γ attenuated HIV-mediated dysregulation of tight junction proteins [53]. Conflicting data may result from tissue difference and further investigation is required.

In endothelial cells, cellular signaling is dependent on two major pathways – PI3K/Akt/mTOR pathway and ERK/MAPK (extracellular signal-regulated kinases/mitogen-activated protein kinases) pathway. VEGF induced endothelial cell proliferation and migration is dependent on the Akt/endothelial synthase pathway [10, 54]. There were conflicting data regarding the mechanism by which rosiglitazone affects various cells including vascular endothelial cells. In our data, rosiglitazone appears to have a role through the PI3K/Akt/mTOR pathway. Accordingly, compared to our study, Goetze et al., who reported conflicting data regarding the effect of rosiglitazone on endothelial cell migration, reported the same results showing that PPAR activators affect endothelial cell migration by targeting Akt [40]. In addition, Wu et al. reported that in HUVECs insulted with high glucose rosiglitazone cellular apoptosis was inhibited through the PI3K/Akt/eNOS pathway [55]. On the contrary, it was suggested that rosiglitazone may influence apoptosis of endothelial progenitor cells under the ERK/MAPK pathway [56] and that rosiglitazone inhibits inflammatory effects by a mechanism involving ERK in human endothelial cells [57]. In ERK/MAPK pathway, the ERK1 and ERK2 MPKs are activated by phosphorylation of threonine and tyrosine residues by the dual specificity kinase MEK1, which induces their translocation into the nucleus where they activate or repress a variety of transcription factors involved in growth and differentiation [58]. The mechanisms by which PPARγ receptor agonists regulate the ERK/MAPK pathway are both direct and indirect mechanisms. The direct involvement of the receptor in mediating TZD action is strongly supported by the results of receptor silencing experiments. However, because receptor silencing does not completely revert TZD effect, a small component of nonreceptorial mechanism cannot be ruled out [57].

It has been reported that PKCβ is activated and selectively inhibits the PI3K / Akt pathway when hyperglycemia and free fatty acid increase in diabetic patients. In Qian Li et al.’s study, they demonstrated that PKCβ overexpression in ApoE^−/−^ mice inhibits Akt activation by insulin, resulting in atherosclerosis [59]. In another study, there is a report that PKCβ has a negative correlation with endothelial insulin signaling in diabetic patients [60].

## Conclusion

There are still conflicts regarding the effect of PPAR-γ agonist on endothelial cell dysfunction. However, PPAR-γ agonist, rosiglitazone might induce endothelial cell migration through the Akt pathway and cause instability of endothelial barrier integrity and cytoskeletal structure, resulting in increased vascular permeability, peripheral edema, and congestive heart failure.

## Abbreviations

DAPI: 4′6-diamidino-2-phenylindole
EB: Evans blue
EBM: endothelial cell basal medium
ECL: enhanced chemiluminescence
EGM: endothelial cell growth medium
ERK/MAPK: extracellular signal-regulated kinases/mitogen-activated protein kinases
FBS: fetal bovine serum
FITC: fluorescein isothiocyanate
HFD: high fat diet
HUVEC: human umbilical vascular endothelial cells
JAM-A: junction adhesion molecule-A
LDL: low density lipoproteins
OCT: optimal cutting temperature
PBS: phosphate-buffered saline
PI3K: phosphatidylinositol-3-kinase
PKC: protein kinase C
PPAR: peroxisome proliferator-activated receptor
TZD: thiazolidinediones
VEGF: vascular endothelial growth factor
VSMC: vascular smooth muscle cell

## References

[1] Ross R (1993). The pathogenesis of atherosclerosis: a perspective for the 1990s. Nature.

[2] Ross R (1999). Atherosclerosis--an inflammatory disease. N Engl J Med.

[3] Kinlay S, Ganz P (1997). Role of endothelial dysfunction in coronary artery disease and implications for therapy. Am J Cardiol.

[4] Lusis AJ (2000). Atherosclerosis. Nature.

[5] Irie S, Tavassoli M (1991). Transendothelial transport of macromolecules: the concept of tissue-blood barriers. Cell Biol Rev.

[6] Stevens T, Garcia JG, Shasby DM, Bhattacharya J, Malik AB (2000). Mechanisms regulating endothelial cell barrier function. Am J Physiol Lung Cell Mol Physiol.

[7] Lum H, Malik AB (1994). Regulation of vascular endothelial barrier function. Am J Phys.

[8] Flamme I, Frolich T, Risau W (1997). Molecular mechanisms of vasculogenesis and embryonic angiogenesis. J Cell Physiol.

[9] Fujio Y, Walsh K (1999). Akt mediates cytoprotection of endothelial cells by vascular endothelial growth factor in an anchorage-dependent manner. J Biol Chem.

[10] Dimmeler S, Dernbach E, Zeiher AM (2000). Phosphorylation of the endothelial nitric oxide synthase at ser-1177 is required for VEGF-induced endothelial cell migration. FEBS Lett.

[11] Rikitake Y, Kawashima S, Yamashita T, Ueyama T, Ishido S, Hotta H, Hirata K, Yokoyama M (2000). Lysophosphatidylcholine inhibits endothelial cell migration and proliferation via inhibition of the extracellular signal-regulated kinase pathway. Arterioscler Thromb Vasc Biol.

[12] Dimmeler S, Zeiher AM (2000). Akt takes center stage in angiogenesis signaling. Circ Res.

[13] Meredith JE, Fazeli B, Schwartz MA (1993). The extracellular matrix as a cell survival factor. Mol Biol Cell.

[14] Dejana E, Valiron O, Navarro P, Lampugnani MG (1997). Intercellular junctions in the endothelium and the control of vascular permeability. Ann N Y Acad Sci.

[15] Lee CH, Olson P, Evans RM (2003). Minireview: lipid metabolism, metabolic diseases, and peroxisome proliferator-activated receptors. Endocrinology.

[16] Kubota N, Terauchi Y, Miki H, Tamemoto H, Yamauchi T, Komeda K, Satoh S, Nakano R, Ishii C, Sugiyama T (1999). PPAR gamma mediates high-fat diet-induced adipocyte hypertrophy and insulin resistance. Mol Cell.

[17] Barak Y, Nelson MC, Ong ES, Jones YZ, Ruiz-Lozano P, Chien KR, Koder A, Evans RM (1999). PPAR gamma is required for placental, cardiac, and adipose tissue development. Mol Cell.

[18] Rosen ED, Sarraf P, Troy AE, Bradwin G, Moore K, Milstone DS, Spiegelman BM, Mortensen RM (1999). PPAR gamma is required for the differentiation of adipose tissue in vivo and in vitro. Mol Cell.

[19] Spiegelman BM (1998). PPAR-gamma: adipogenic regulator and thiazolidinedione receptor. Diabetes.

[20] Marx N, Bourcier T, Sukhova GK, Libby P, Plutzky J (1999). PPARgamma activation in human endothelial cells increases plasminogen activator inhibitor type-1 expression: PPARgamma as a potential mediator in vascular disease. Arterioscler Thromb Vasc Biol.

[21] Duan SZ, Usher MG, Mortensen RM (2008). Peroxisome proliferator-activated receptor-gamma-mediated effects in the vasculature. Circ Res.

[22] Nesto RW, Bell D, Bonow RO, Fonseca V, Grundy SM, Horton ES, Le Winter M, Porte D, Semenkovich CF, Smith S (2003). Thiazolidinedione use, fluid retention, and congestive heart failure: a consensus statement from the American Heart Association and American Diabetes Association. October 7, 2003. Circulation.

[23] Sinagra T, Tamburella A, Urso V, Siarkos I, Drago F, Bucolo C, Salomone S (2013). Reversible inhibition of vasoconstriction by thiazolidinediones related to PI3K/Akt inhibition in vascular smooth muscle cells. Biochem Pharmacol.

[24] Prusty D, Park BH, Davis KE, Farmer SR (2002). Activation of MEK/ERK signaling promotes adipogenesis by enhancing peroxisome proliferator-activated receptor gamma (PPARgamma ) and C/EBPalpha gene expression during the differentiation of 3T3-L1 preadipocytes. J Biol Chem.

[25] Naruse K, Rask-Madsen C, Takahara N, Ha SW, Suzuma K, Way KJ, Jacobs JR, Clermont AC, Ueki K, Ohshiro Y (2006). Activation of vascular protein kinase C-beta inhibits Akt-dependent endothelial nitric oxide synthase function in obesity-associated insulin resistance. Diabetes.

[26] Luconi M, Mangoni M, Gelmini S, Poli G, Nesi G, Francalanci M, Pratesi N, Cantini G, Lombardi A, Pepi M (2010). Rosiglitazone impairs proliferation of human adrenocortical cancer: preclinical study in a xenograft mouse model. Endocr Relat Cancer.

[27] Patel SS, Gupta S, Udayabanu M (2016). Urtica Dioica modulates hippocampal insulin signaling and recognition memory deficit in streptozotocin induced diabetic mice. Metab Brain Dis.

[28] Sugii S, Olson P, Sears DD, Saberi M, Atkins AR, Barish GD, Hong SH, Castro GL, Yin YQ, Nelson MC (2009). PPARgamma activation in adipocytes is sufficient for systemic insulin sensitization. Proc Natl Acad Sci U S A.

[29] He W, Barak Y, Hevener A, Olson P, Liao D, Le J, Nelson M, Ong E, Olefsky JM, Evans RM (2003). Adipose-specific peroxisome proliferator-activated receptor gamma knockout causes insulin resistance in fat and liver but not in muscle. Proc Natl Acad Sci U S A.

[30] Li J, Davies BR, Han S, Zhou M, Bai Y, Zhang J, Xu Y, Tang L, Wang H, Liu YJ (2013). The AKT inhibitor AZD5363 is selectively active in PI3KCA mutant gastric cancer, and sensitizes a patient-derived gastric cancer xenograft model with PTEN loss to Taxotere. J Transl Med.

[31] Cherrin C, Haskell K, Howell B, Jones R, Leander K, Robinson R, Watkins A, Bilodeau M, Hoffman J, Sanderson P (2010). An allosteric Akt inhibitor effectively blocks Akt signaling and tumor growth with only transient effects on glucose and insulin levels in vivo. Cancer Biol Ther.

[32] Inoue M, Itoh H, Tanaka T, Chun TH, Doi K, Fukunaga Y, Sawada N, Yamshita J, Masatsugu K, Saito T (2001). Oxidized LDL regulates vascular endothelial growth factor expression in human macrophages and endothelial cells through activation of peroxisome proliferator-activated receptor-gamma. Arterioscler Thromb Vasc Biol.

[33] Law RE, Goetze S, Xi XP, Jackson S, Kawano Y, Demer L, Fishbein MC, Meehan WP, Hsueh WA (2000). Expression and function of PPARgamma in rat and human vascular smooth muscle cells. Circulation.

[34] Yang T, Soodvilai S (2008). Renal and vascular mechanisms of thiazolidinedione-induced fluid retention. PPAR Res.

[35] Schram MT, Stehouwer CD (2005). Endothelial dysfunction, cellular adhesion molecules and the metabolic syndrome. Horm Metab Res.

[36] Marx N, Mach F, Sauty A, Leung JH, Sarafi MN, Ransohoff RM, Libby P, Plutzky J, Luster AD (2000). Peroxisome proliferator-activated receptor-gamma activators inhibit IFN-gamma-induced expression of the T cell-active CXC chemokines IP-10, Mig, and I-TAC in human endothelial cells. J Immunol.

[37] Pasceri V, Wu HD, Willerson JT, Yeh ET (2000). Modulation of vascular inflammation in vitro and in vivo by peroxisome proliferator-activated receptor-gamma activators. Circulation.

[38] Jackson SM, Parhami F, Xi XP, Berliner JA, Hsueh WA, Law RE, Demer LL (1999). Peroxisome proliferator-activated receptor activators target human endothelial cells to inhibit leukocyte-endothelial cell interaction. Arterioscler Thromb Vasc Biol.

[39] Chen NG, Sarabia SF, Malloy PJ, Zhao XY, Feldman D, Reaven GM (1999). PPARgamma agonists enhance human vascular endothelial adhesiveness by increasing ICAM-1 expression. Biochem Biophys Res Commun.

[40] Goetze S, Eilers F, Bungenstock A, Kintscher U, Stawowy P, Blaschke F, Graf K, Law RE, Fleck E, Grafe M (2002). PPAR activators inhibit endothelial cell migration by targeting Akt. Biochem Biophys Res Commun.

[41] Xin X, Yang S, Kowalski J, Gerritsen ME (1999). Peroxisome proliferator-activated receptor gamma ligands are potent inhibitors of angiogenesis in vitro and in vivo. J Biol Chem.

[42] Murata T, Hata Y, Ishibashi T, Kim S, Hsueh WA, Law RE, Hinton DR (2001). Response of experimental retinal neovascularization to thiazolidinediones. Arch Ophthalmol.

[43] Terrasi M, Bazan V, Caruso S, Insalaco L, Amodeo V, Fanale D, Corsini LR, Contaldo C, Mercanti A, Fiorio E (2013). Effects of PPARgamma agonists on the expression of leptin and vascular endothelial growth factor in breast cancer cells. J Cell Physiol.

[44] Werner C, Kamani CH, Gensch C, Bohm M, Laufs U (2007). The peroxisome proliferator-activated receptor-gamma agonist pioglitazone increases number and function of endothelial progenitor cells in patients with coronary artery disease and normal glucose tolerance. Diabetes.

[45] Celletti FL, Waugh JM, Amabile PG, Brendolan A, Hilfiker PR, Dake MD (2001). Vascular endothelial growth factor enhances atherosclerotic plaque progression. Nat Med.

[46] Chintalgattu V, Harris GS, Akula SM, Katwa LC (2007). PPAR-gamma agonists induce the expression of VEGF and its receptors in cultured cardiac myofibroblasts. Cardiovasc Res.

[47] Kanata S, Akagi M, Nishimura S, Hayakawa S, Yoshida K, Sawamura T, Munakata H, Hamanishi C (2006). Oxidized LDL binding to LOX-1 upregulates VEGF expression in cultured bovine chondrocytes through activation of PPAR-gamma. Biochem Biophys Res Commun.

[48] Schiefelbein D, Seitz O, Goren I, Dissmann JP, Schmidt H, Bachmann M, Sader R, Geisslinger G, Pfeilschifter J, Frank S (2008). Keratinocyte-derived vascular endothelial growth factor biosynthesis represents a pleiotropic side effect of peroxisome proliferator-activated receptor-gamma agonist troglitazone but not rosiglitazone and involves activation of p38 mitogen-activated protein kinase: implications for diabetes-impaired skin repair. Mol Pharmacol.

[49] Whiteside C, Wang H, Xia L, Munk S, Goldberg HJ, Fantus IG (2009). Rosiglitazone prevents high glucose-induced vascular endothelial growth factor and collagen IV expression in cultured mesangial cells. Exp Diabetes Res.

[50] Mattos RT, Bosco AA, Nogueira-Machado JA (2012). Rosiglitazone, a PPAR-gamma agonist, inhibits VEGF secretion by peripheral blood mononuclear cells and ROS production by human leukocytes. Inflamm Res.

[51] Gavard J, Gutkind JS (2006). VEGF controls endothelial-cell permeability by promoting the beta-arrestin-dependent endocytosis of VE-cadherin. Nat Cell Biol.

[52] Ogasawara N, Kojima T, Go M, Ohkuni T, Koizumi J, Kamekura R, Masaki T, Murata M, Tanaka S, Fuchimoto J (2010). PPARgamma agonists upregulate the barrier function of tight junctions via a PKC pathway in human nasal epithelial cells. Pharmacol Res.

[53] Huang W, Eum SY, Andras IE, Hennig B, Toborek M (2009). PPARalpha and PPARgamma attenuate HIV-induced dysregulation of tight junction proteins by modulations of matrix metalloproteinase and proteasome activities. FASEB J.

[54] Morales-Ruiz M, Fulton D, Sowa G, Languino LR, Fujio Y, Walsh K, Sessa WC (2000). Vascular endothelial growth factor-stimulated actin reorganization and migration of endothelial cells is regulated via the serine/threonine kinase Akt. Circ Res.

[55] Wu J, Lei MX, Xie XY, Liu L, She YM, Mo J, Wang S (2009). Rosiglitazone inhibits high glucose-induced apoptosis in human umbilical vein endothelial cells through the PI3K/Akt/eNOS pathway. Can J Physiol Pharmacol.

[56] Xu S, Zhao Y, Yu L, Shen X, Ding F, Fu G (2011). Rosiglitazone attenuates endothelial progenitor cell apoptosis induced by TNF-alpha via ERK/MAPK and NF-kappaB signal pathways. J Pharmacol Sci.

[57] Lombardi A, Cantini G, Piscitelli E, Gelmini S, Francalanci M, Mello T, Ceni E, Varano G, Forti G, Rotondi M (2008). A new mechanism involving ERK contributes to rosiglitazone inhibition of tumor necrosis factor-alpha and interferon-gamma inflammatory effects in human endothelial cells. Arterioscler Thromb Vasc Biol.

[58] Seger R, Krebs EG (1995). The MAPK signaling cascade. FASEB J.

[59] Li Q, Park K, Li C, Rask-Madsen C, Mima A, Qi W, Mizutani K, Huang P, King GL (2013). Induction of vascular insulin resistance and endothelin-1 expression and acceleration of atherosclerosis by the overexpression of protein kinase C-beta isoform in the endothelium. Circ Res.

[60] Tabit CE, Shenouda SM, Holbrook M, Fetterman JL, Kiani S, Frame AA, Kluge MA, Held A, Dohadwala MM, Gokce N (2013). Protein kinase C-beta contributes to impaired endothelial insulin signaling in humans with diabetes mellitus. Circulation.

